# Co-Production of Nattokinase and Menaquinone-7 by *Bacillus subtilis* Natto: Metabolic Trade-Offs, Regulatory Mechanisms, and Dynamic Fermentation Strategies

**DOI:** 10.3390/foods15213869

**Published:** 2026-10-29

**Authors:** Yuan Hou, Pengchao Wang

**Affiliations:** 1 Aulin College, Northeast Forestry University, Harbin 150040, China; alan2024214660@nefu.edu.cn; 2 Heilongjiang Provincial Key Laboratory of Forest Sustainable Management and Environmental Microbial Engineering, Northeast Forestry University, Harbin 150040, China

**Keywords:** *Bacillus subtilis* natto, nattokinase, menaquinone-7, vitamin K2, metabolic trade-off, dynamic fermentation, multi-omics, functional foods

## Abstract

*Bacillus subtilis* natto can co-produce nattokinase (NK) and menaquinone-7 (MK-7), but their distinct biosynthetic demands, cellular localization, and production kinetics may be associated with condition-dependent trade-offs. This review critically evaluates the biosynthetic mechanisms and multi-omics regulatory characteristics of NK and MK-7 and examines how oxygen-related conditions, substrate composition, and redox state are associated with co-production performance. Dynamic two-stage fermentation has been proposed as a promising, system-dependent framework for coordinating the different production phases of NK and MK-7. However, current evidence remains limited to specific strains, substrates, and reactor configurations. Future studies should integrate ^13^C metabolic flux analysis, real-time sensing, standardized detection, and non-GM strain improvement to drive NK-MK-7 co-production from empirical optimization toward mechanism-driven predictive design, providing a theoretical and technical basis for dual-functional foods and multi-product fermentation.

## 1. Introduction

Fermented foods have served humankind for thousands of years, initially as a means of preservation and now widely recognized for their multiple contributions to nutritional enhancement, flavor development, and health promotion. The microbial biotransformation of raw materials can not only enhance the bioavailability of nutrients and generate physiologically active secondary metabolites, but can also create distinctive sensory characteristics that define regional food cultures. Among fermented foods, natto—a traditional Japanese food made from steamed soybeans fermented by *Bacillus subtilis* natto—occupies a unique position. It is not only an important traditional food but also a natural source of metabolites including NK and MK-7, which have been investigated for fibrinolytic activity and vitamin-K-related nutritional functions, respectively [1,2,3,4]. Its starter microorganism, *Bacillus subtilis* natto, is an exemplary “dual-functional” food fermentation starter: a single bacterial strain can simultaneously produce an extracellular enzyme (nattokinase, NK) and an intracellular nutritional metabolite (menaquinone-7, MK-7), each with independent and distinct functional relevance.

Nattokinase (NK) is a secreted serine protease that has attracted considerable research interest because of its fibrinolytic activity. It was first isolated and identified from traditional natto by Sumi et al. in 1987 [5,6]. Structurally, NK belongs to the subtilisin family, and its biological properties have mainly been investigated based on its ability to degrade fibrin in vitro and its potential effects associated with fibrinolytic regulation [7,8,9]. As an extracellular enzyme, NK represents a distinctive type of functional metabolite produced by *Bacillus subtilis* natto, in which protein synthesis, secretion efficiency, and extracellular stability collectively determine the final measurable activity. Therefore, NK production is not only related to enzyme expression but also depends on the physiological state of the producing strain and the surrounding fermentation environment.

MK-7 is a long-chain menaquinone homologue belonging to the vitamin K2 family and represents one of the characteristic functional components of natto [10]. Compared with other vitamin K homologues, MK-7 exhibits relatively longer persistence in circulation and favorable oral utilization characteristics, which has contributed to its extensive investigation as a nutritional component [11,12]. MK-7 has been studied in relation to extrahepatic vitamin K status and selected biomarkers associated with bone and vascular physiology [13,14,15]. During natto fermentation, MK-7 is the major menaquinone homologue synthesized by *Bacillus subtilis* natto. Unlike the extracellularly secreted NK, MK-7 is synthesized intracellularly and is mainly retained in the bacterial cell membrane, where it functions as an electron carrier involved in respiratory processes [16]. This distinct cellular localization indicates that NK and MK-7 may experience different production dynamics during fermentation.

Simultaneous production of NK and MK-7 in a single fermentation process represents an attractive strategy for developing multifunctional fermented foods, concentrated fermentates, or fractionated functional ingredients. Such a co-production approach may enable the generation of multiple functional outputs from a shared microbial fermentation platform and provide opportunities for integrated process optimization and downstream processing. From a practical processing perspective, the distinct localization of extracellular NK and cell-associated MK-7 may facilitate sequential product recovery; Yi et al. reported recovery efficiencies of 77.1% for NK and 75.4% for MK-7 from a single *B. subtilis* natto fermentation system [17]. However, most previous studies have focused on optimizing NK or MK-7 production separately, and the basis for their coordinated production remains insufficiently understood. Differences in biosynthetic characteristics, cellular localization, and physiological requirements may contribute to potential production constraints, which require further investigation.

To date, only a limited number of studies have directly explored NK-MK-7 co-production. These studies have provided preliminary evidence that coordinated improvement of NK and MK-7 production may be achieved through different strategies, including fermentation-condition optimization, metabolic flux redistribution, and spatially differentiated product recovery [18,19,20]. However, whether these approaches can be generalized across different strains and fermentation systems remains unclear.

Literature for this critical review was identified through searches of Web of Science, PubMed, ScienceDirect, Google Scholar, and the China National Knowledge Infrastructure (CNKI), conducted between March and September 2026 and covering publications from 1987 to 2026. Peer-reviewed studies and authoritative technical or regulatory sources directly relevant to NK and/or MK-7 biosynthesis, production, co-production, fermentation engineering, analytical methods, safety, and regulation were prioritized, whereas clearly unrelated studies were excluded.

This review critically evaluates evidence for coordinated nattokinase (NK) and menaquinone-7 (MK-7) production by *Bacillus subtilis* natto and related *B. subtilis* strains, extending previous reviews that have largely addressed NK or MK-7 separately. Particular attention is given to studies that quantify both products within the same fermentation system, while evidence derived from single-product optimization, strain screening, food matrix fermentation, metabolic engineering, and downstream fractionation is discussed separately. The review further examines whether reported differences in oxygen demand, metabolic state, product localization, and production kinetics support a rationale for physiology-guided dynamic fermentation, with the broader aim of improving or appropriately balancing both outputs and informing their potential translation into dual-functional fermented foods or functional ingredients.

## 2. Biosynthetic Pathways and Metabolic Competition

### 2.1. Nattokinase: Biosynthesis and Secretion of an Extracellular Protease

Nattokinase (NK), also referred to as subtilisin NAT, is a secreted serine protease associated with the aprN locus in natto strains. In *B. subtilis* natto BEST195, aprN is orthologous to the canonical aprE locus of *B. subtilis* 168 and shares approximately 99.2% nucleotide-sequence similarity; therefore, aprN should not automatically be interpreted as a distinct natto-specific paralog in cross-strain comparisons [21]. NK is synthesized as a pre-proenzyme. An N-terminal signal peptide directs the unfolded polypeptide to the Sec secretion system; after transmembrane transport, the signal peptide is cleaved, and the propeptide acts as an intramolecular chaperone to promote correct folding and further maturation into the active enzyme [22,23]. Therefore, NK production depends not only on transcriptional and translational processes but also on Sec-mediated secretion efficiency and subsequent protein maturation.

Extracellular protease expression is linked to the regulatory network governing the transition of cells from rapid growth to stationary phase. The DegS-DegU system directly participates in *aprE* promoter regulation [24]; CodY and ScoC connect nitrogen sources, branched-chain amino acids, and overall nutritional status to protease gene expression [25]. ComQXPA quorum sensing can influence extracellular protease formation through DegQ [26], while the RelA stringent response and SalA-ScoC regulation also alter *aprE* expression [27,28]. These regulatory relationships indicate that high NK production generally requires an intermediate physiological state in which sufficient peptides and energy are available but population-density or nutrient-transition signals have already emerged, rather than unrestricted elevation of any single nutrient.

SecA-mediated protein transport requires ATP and is coupled to the transmembrane proton motive force, indicating that large-scale NK secretion imposes an additional energetic demand on the producing cell [22]. For NK-MK-7 co-production, the extracellular localization of NK introduces an additional consideration: extending fermentation may favor MK-7 accumulation but may also increase the exposure time of secreted NK to environmental changes and potential activity loss. NK activity is affected by fermentation conditions, including pH and other physicochemical factors, which may influence the final measurable enzyme activity [23].

### 2.2. Menaquinone-7: Biosynthesis of a Membrane-Associated Vitamin

MK-7 consists of a naphthoquinone ring and seven isoprenyl units, is mainly embedded in the cytoplasmic membrane, and participates in respiratory electron transfer. Its naphthoquinone backbone derives from chorismate, the end product of the shikimate pathway: MenF first forms isochorismate, after which MenD and other enzymes advance the pathway to 1,4-dihydroxy-2-naphthoic acid (DHNA). Early genetic studies established the organization and regulation of the menaquinone gene cluster in *Bacillus subtilis* [29,30], and structural and catalytic studies of MenD further support its role as a key control node [31].

The side chain is supplied by the MEP pathway in the form of IPP and DMAPP, with starting substrates derived mainly from pyruvate and glyceraldehyde-3-phosphate. This pathway contains multiple reduction reactions and is sensitive to NAD(P)H supply. Enhancement of precursor-generating steps such as DXS can increase MK-7 synthesis [32], and modular coordination of the MEP and menaquinone modules can further increase production [33]. Finally, the membrane protein MenA attaches the isoprenoid side chain to a DHNA derivative, and MenG completes methylation; membrane capacity, electron transfer, and membrane homeostasis therefore become constraints as important as “enzyme amount.” Improved MK-7 accumulation through membrane-lipid and electron-transfer engineering has been validated [34], and coordinated regulation of MenD expression and MenA stability has also achieved relatively high titers [35].

Biofilms can alter cell density, membrane area, oxygen diffusion, and local redox state and are therefore often associated with MK-7 production [36]. In addition, Peng et al. found that under different agitation conditions, a higher agitation speed of 200 rpm increased MK-7 production to 18.56 mg/L, approximately twice that achieved at 100 rpm, accompanied by an approximately 3.49-fold upregulation of *menD* expression [37,38]. Agitation should be considered a composite process variable because it simultaneously modifies gas–liquid mass transfer, mixing, shear exposure, and the cellular microenvironment. The coordinated changes in MK-7 production and menD expression therefore highlight the sensitivity of MK-7 biosynthesis to the fermentation state created by reactor operation, supporting the use of process-state regulation rather than a single oxygen-based criterion in fermentation optimization.

Chorismate is an important precursor in MK-7 biosynthesis, serving as the starting material for the formation of DHNA through the menaquinone pathway. In addition to its role in menaquinone biosynthesis, chorismate also participates in the synthesis of aromatic amino acids, including phenylalanine, tyrosine, and tryptophan. Therefore, chorismate availability may directly affect precursor supply for MK-7 biosynthesis, whereas its potential influence on NK production is indirect, through aromatic amino acid biosynthesis and subsequent protein synthesis.

### 2.3. Metabolic and Process-Level Trade-Offs in Co-Production

In this review, “trade-off” is used as an umbrella term for condition-dependent constraints in co-production and does not necessarily imply direct precursor competition. Direct evidence refers to a phenomenon measured within the same experimental system, whereas indirect evidence is inferred from related single-product, physiological, or omics studies. Mechanistic hypotheses denote biologically plausible relationships that have not yet been quantitatively validated.

The first major challenge in NK-MK-7 co-production is the allocation of cellular carbon flux among product biosynthesis, competing metabolic pathways, and cellular maintenance. During fermentation, carbon resources must be distributed not only toward NK and MK-7 formation but also toward growth-associated metabolism and overflow pathways that reduce precursor availability. Therefore, improving co-production efficiency requires understanding how carbon flux is redirected among competing metabolic demands. A representative example is the *bdhA* knockout study reported by Huang et al. (2024), in which reduced 2,3-butanediol formation was accompanied by increased measured NK activity and MK-7 concentration [19]. This result is consistent with the possibility that reducing overflow metabolism can increase the availability of cellular resources for both outputs. However, because *bdhA* deletion affects central metabolism broadly, the observed phenotype does not establish the specific carbon flux routes or precursor pools responsible for the improvement. In particular, competition involving chorismate allocation between menaquinone biosynthesis and aromatic amino acid synthesis remains a mechanistic hypothesis that requires quantitative validation using isotope tracing and metabolic-flux analysis.

The second dimension of trade-off involves cellular energy metabolism and redox balance. NK production requires substantial investment in protein synthesis and Sec-dependent secretion, which relies on ATP consumption and the proton motive force to transport and mature extracellular enzymes. Therefore, high-level NK production may impose an additional energetic burden on the cell, potentially affecting the allocation of resources toward other biosynthetic processes. In contrast, MK-7 biosynthesis is closely linked to membrane metabolism and respiratory activity. The formation of its isoprenoid side chain through the MEP pathway requires reducing equivalents, while MK-7 itself functions as an electron carrier within the respiratory chain, making its accumulation dependent on cellular redox status and membrane homeostasis. Consequently, environmental conditions that favor rapid respiration and ATP generation may not always be optimal for MK-7 accumulation, whereas conditions supporting quinone metabolism may impose different constraints on NK secretion. Transcriptomic studies under different oxygen conditions further suggest that NK secretion-related genes and menaquinone biosynthetic genes respond differently to oxygen availability, indicating condition-dependent regulation of the two production processes [37,38].

The third dimension of trade-off is associated with cellular compartmentalization and physiological state. Unlike MK-7, which is mainly retained within the cytoplasmic membrane as a respiratory quinone, NK is secreted into the extracellular environment where its accumulation and stability are influenced by extracellular conditions. Prolonged fermentation may therefore create different consequences for the two products: continued cultivation can promote membrane-associated MK-7 accumulation through increased biomass formation and respiratory activity, while extracellular NK may experience gradual activity loss due to environmental exposure, degradation, or unfavorable fermentation conditions.

In addition to product localization, physiological transitions of *Bacillus subtilis* natto can strongly affect co-production performance. Differences between wild-type and domesticated strains in γ-PGA production, biofilm formation, and surface properties indicate that cellular organization influences metabolite distribution and fermentation behavior [39]. Moreover, DegQ-related regulation affects γ-PGA-associated phenotypes [40], while biofilms provide spatially heterogeneous microenvironments that can generate distinct metabolic states within the population [41]. Regulation of MK-7 metabolism through Spo0A-associated quorum-sensing mechanisms further demonstrates that developmental state and population-level regulation can influence menaquinone production [42].

**Figure 1:**
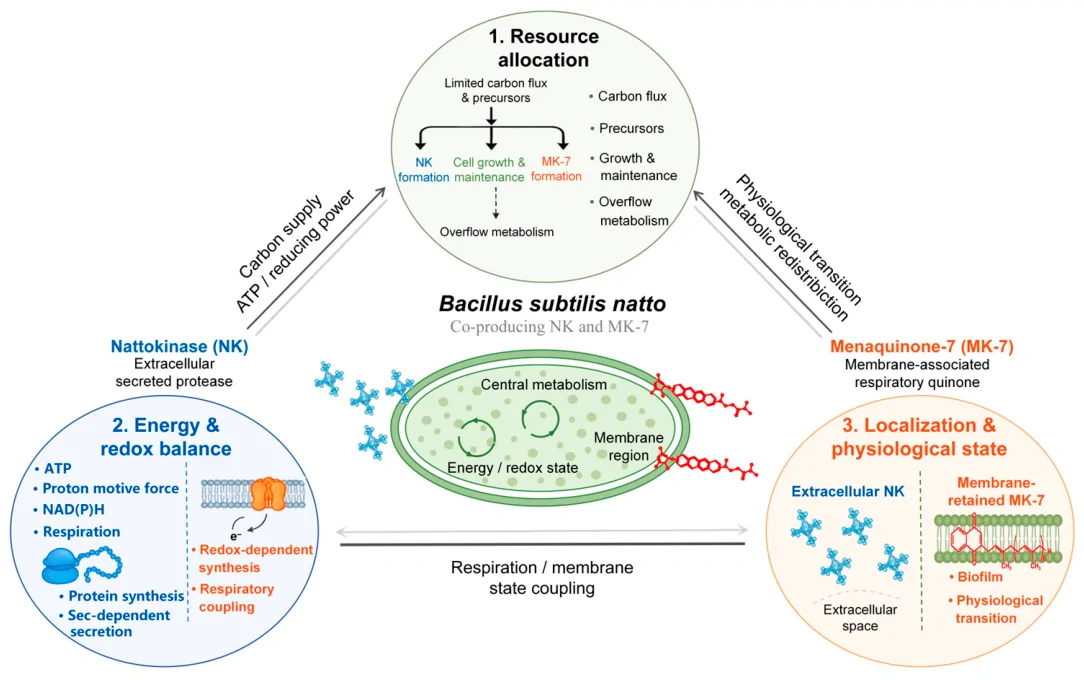
Conceptual framework illustrating metabolic and process-level trade-offs between nattokinase (NK) and menaquinone-7 (MK-7) co-production in *Bacillus subtilis* natto. NK and MK-7 differ in biosynthetic requirements, cellular localization, and physiological dependence, resulting in multiple layers of potential trade-offs during co-production. Carbon allocation, energy and redox balance, and cellular compartmentalization represent major factors influencing the distribution of resources between extracellular NK secretion and membrane-associated MK-7 formation.

As summarized in Figure 1, NK and MK-7 differ substantially in biosynthetic location, cellular compartmentalization, and physiological dependence, which together create multiple layers of trade-offs during co-production. The major metabolic and process requirements associated with each product are further compared in Table 1. These differences provide a mechanistic basis for considering dynamic fermentation strategies that adjust environmental conditions according to the different production requirements of NK and MK-7.

## 3. Multi-Omics-Guided Regulatory Coupling Mechanisms

**Figure 2:**
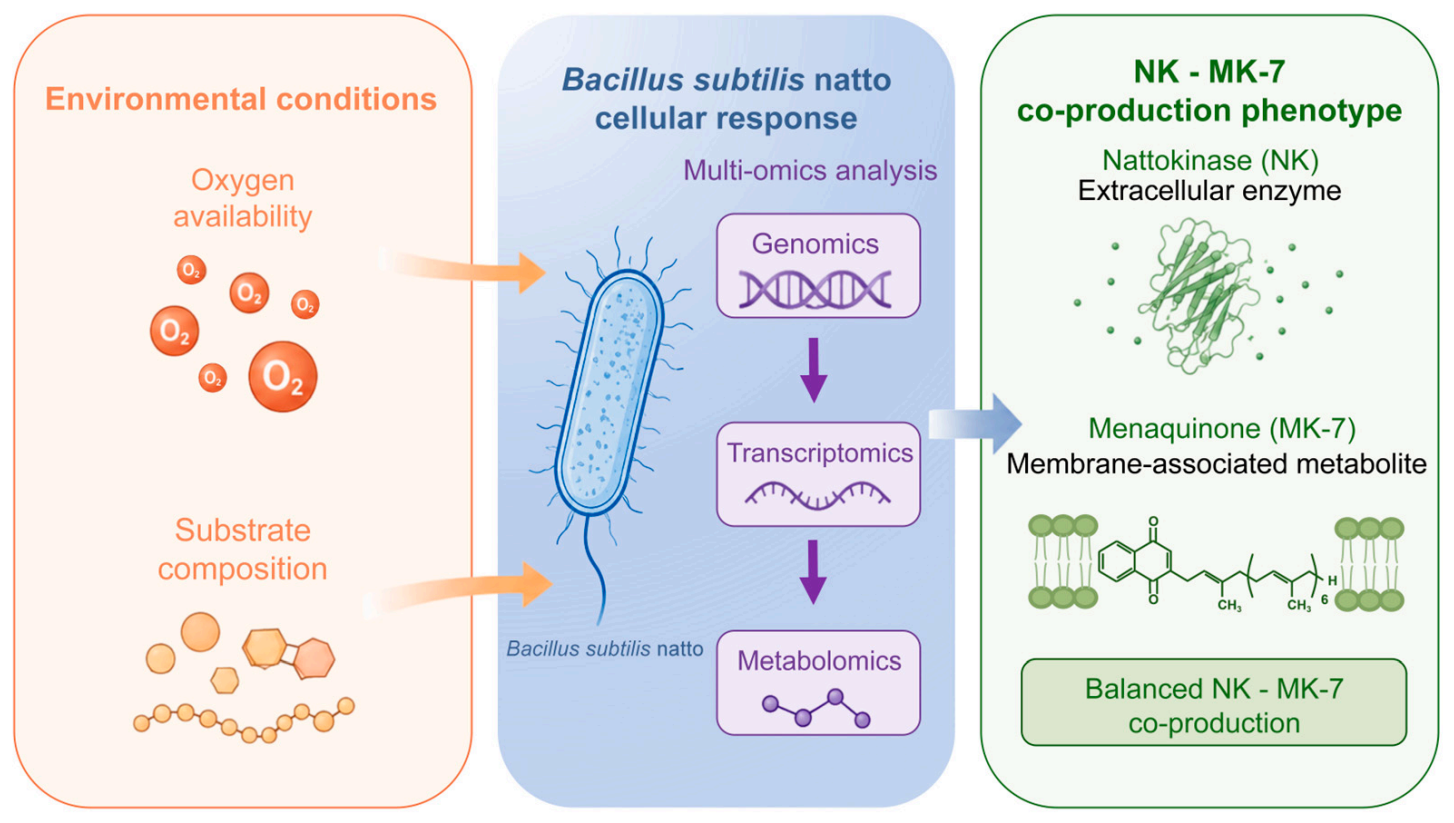
A multi-omics framework linking environmental responses to the regulation of nattokinase (NK) and menaquinone-7 (MK-7) co-production in *Bacillus subtilis* natto. Environmental factors, including oxygen availability and substrate composition, influence cellular regulatory states. Multi-omics approaches integrating genomics, transcriptomics, and metabolomics provide hierarchical insights into genetic characteristics, regulatory responses, and metabolic adjustments, thereby improving the understanding of balanced NK-MK-7 co-production.

The value of multi-omics lies in transforming the empirical observation that “a given condition produces a higher yield” into testable regulatory hypotheses. This section examines how omics approaches clarify the genetic basis of the natto phenotype, reveal condition-dependent transcriptional and metabolic responses underlying flux redistribution, and guide metabolic engineering strategies that convert candidate nodes into verifiable perturbations to enhance co-production. The conceptual relationship among environmental conditions, multi-omics analyses, and NK-MK-7 co-production regulation is summarized in Figure 2.

### 3.1. Genomic Basis of the Natto Phenotype

Not all *Bacillus subtilis* strains possess the genetic capacity required for natto fermentation, indicating that natto-associated traits are determined by complex strain-specific genomic architectures rather than by a single marker gene. Classical screening studies showed that γ-PGA positivity alone is insufficient to predict desirable natto characteristics, including hardness, surface coverage, stringiness, and odor, whereas biotin auxotrophy exhibits a stronger association with natto adaptability [1]. These findings suggest that multiple physiological processes, including extracellular matrix formation, nutrient utilization, and cellular adaptation, collectively contribute to the natto phenotype.

Comparative genomic analyses further revealed that natto-producing strains generally share a conserved core genome but differ substantially in accessory genomic regions. BEST195 and laboratory strain 168 exhibit high genomic similarity, yet functional differences have been identified in regions associated with degQ, swrAA, ComQXP signaling, and mobile genetic elements, which may influence biofilm formation, regulatory responses, and surface-associated characteristics [2]. In addition, isolates derived from different non-salted legume fermentations display diversity in loci related to biotin metabolism, γ-PGA production, protease activity, and secondary metabolism, indicating that adaptation to fermentation environments involves coordinated regulation of multiple functional modules [3,4]. Comparative genomic analysis by Kamada et al. (2015) further confirmed the existence of a conserved natto core genome accompanied by extensive accessory-gene variation, highlighting the genetic diversity underlying strain-specific fermentation properties [3].

These genomic differences provide an important basis for understanding why different natto strains exhibit distinct capabilities in extracellular enzyme secretion and metabolite production. In the context of NK-MK-7 co-production, strain-level genomic characterization is therefore essential, because variations in regulatory networks, extracellular matrix formation, and metabolic capacity may influence both NK secretion efficiency and MK-7 biosynthetic performance.

### 3.2. Transcriptomic Responses to Fermentation Conditions

Transcriptomic analyses provide an important approach for understanding how fermentation conditions reshape cellular regulatory states. Rather than acting merely as passive nutrient sources, fermentation matrices and environmental parameters can influence transcriptional programs associated with nutrient acquisition, stress adaptation, secretion capacity, and metabolic allocation. However, transcriptomic responses should be interpreted as regulatory signals rather than direct measurements of metabolic flux or product formation.

Substrate composition represents one of the major factors influencing transcriptional states during fermentation. When Liu et al. compared different fermentation substrates, soybean and sunflower kernels supported 9582 FU/mL and 7466 FU/mL NK production, respectively, whereas no detectable NK activity was observed in the Lycium barbarum matrix under their experimental conditions. Transcriptomic analysis suggested that protein-rich substrates enhanced peptide transport systems, central metabolic activity, and *aprE*-related expression, providing molecular clues for the substrate-dependent differences in NK production [43]. Another RNA-seq study further associated γ-PGA formation, extracellular protease secretion, and stationary-phase regulation, suggesting that NK production is governed by coordinated physiological transitions rather than by activation of a single biosynthetic gene alone [44].

These findings indicate that substrate optimization influences NK production not only through nutrient availability but also through broader regulatory responses. In particular, extracellular enzyme production in *Bacillus subtilis* natto is closely associated with the transition from growth-associated metabolism toward stationary-phase physiological states. Therefore, transcriptomic analysis can help identify regulatory states favorable for NK production, although transcript abundance alone cannot fully predict final enzyme activity because protein production, secretion efficiency, extracellular stability, and fermentation conditions also contribute to the measurable phenotype. Proteomic or secretome analysis combined with enzyme-activity measurements could therefore provide a useful bridge between transcriptional regulation and functional NK production.

Oxygen-related fermentation conditions represent another important environmental factor affecting transcriptional responses, as changes in the fermentation environment can influence respiration, oxidative stress, energy metabolism, and membrane physiology. Peng et al. investigated MK-7 production under different agitation conditions and observed that higher agitation was associated with enhanced MK-7 accumulation and related gene-expression changes under their experimental system [38]. These findings further indicate that MK-7 regulation is condition-dependent and responsive to the physiological and regulatory state established under different fermentation conditions.

More recent evidence further illustrates this system dependence. Yi et al. reported a 199.4% increase in MK-7 yield when soy protein hydrolysates were combined with a two-stage aeration strategy, accompanied by substantial changes in biofilm formation and cell viability [45]. Because nutrient composition, biofilm physiology, and aeration were altered together, the observed improvement should be interpreted as a process-level response rather than an oxygen-specific causal rule. Moreover, transcriptional changes should not be directly equated with increased metabolic flux or product accumulation. Oxygen transfer efficiency, agitation behavior, reactive oxygen species (ROS) generation, substrate consumption, and membrane physiological state are simultaneously affected during fermentation. Therefore, transcriptomic studies provide valuable regulatory clues for identifying condition-dependent responses, but integration with metabolomic analysis, metabolic flux measurements, and fermentation phenotyping is required to determine whether transcriptional changes translate into improved NK-MK-7 co-production performance.

### 3.3. Metabolomic Analysis of Flux Redistribution

Metabolomics provides a closer connection to fermentation phenotypes by capturing changes in intracellular and extracellular metabolite profiles. Compared with transcriptomic analysis, which primarily reflects regulatory responses, metabolomic approaches provide additional information regarding the biochemical consequences of environmental changes and genetic interventions. However, current metabolomic studies in *Bacillus subtilis* natto mainly reveal relative abundance changes rather than quantitative metabolic flux and therefore should be interpreted as evidence of metabolic redistribution rather than direct proof of carbon-flow allocation.

Yin et al. performed widely targeted metabolomic analysis of natto fermentation and detected 569 metabolites, including differential changes associated with amino acid, organic acid, lipid, and flavor metabolism [46]. These results demonstrate that fermentation involves extensive metabolic remodeling beyond individual product pathways. However, the metabolites most relevant to NK-MK-7 co-production, including chorismate, DHNA, and isoprenoid precursors such as IPP/DMAPP, generally lack absolute quantification and time-resolved measurements. Therefore, a complete carbon flux balance between NK synthesis, MK-7 biosynthesis, and competing cellular pathways cannot yet be directly established.

The *bdhA* knockout study provides an important example of how metabolomic information can be combined with transcriptomic and fermentation data to interpret co-production improvement. Huang et al. showed that deletion of *bdhA* reduced 2,3-butanediol overflow metabolism and was associated with enhanced NK and MK-7 production [19]. These results show an association between reduced 2,3-butanediol formation and increased NK and MK-7 production. However, the available data do not resolve the intracellular flux redistribution responsible for this phenotype and therefore do not demonstrate direct precursor competition between the NK and MK-7 biosynthetic pathways.

Metabolomic analysis has also highlighted the importance of physiological state during fermentation. Changes in organic acids, amino acids, and energy-related metabolites may reflect transitions between growth, stress adaptation, stationary-phase metabolism, and product accumulation. Such information can provide useful indicators for identifying favorable fermentation states. Nevertheless, metabolite abundance represents the combined outcome of synthesis, consumption, transport, and degradation processes; therefore, changes in metabolite levels cannot be directly interpreted as increased pathway flux.

To establish a more quantitative understanding of metabolic trade-offs, future studies should integrate metabolomics with isotope-labeling approaches, such as ^13^C metabolic flux analysis (^13^C-MFA), together with transcriptomic and fermentation-process data. Such integrated strategies may help distinguish whether improved NK-MK-7 co-production results from enhanced pathway capacity, reduced competing metabolism, or broader physiological adaptation.

### 3.4. Omics-Guided Engineering: From Correlation to Causality

The core of omics-guided engineering is to convert correlated nodes into reversible, verifiable perturbations. In recent years, combinatorial metabolic engineering through regulation of precursor supply, competing branches, and cofactors enabled engineered *B. subtilis* to reach 474 mg/L MK-7 at the 50 L scale, indicating that the theoretical upper limit of pathway engineering can still be substantially increased [47]; however, their performance cannot be directly extrapolated to food-grade natto starters in terms of sensory, safety, and genetic-stability requirements. Food-derived strains reached 23.43 mg/L MK-7 through screening and fed-batch cultivation [48], while non-GM mutant strains obtained by ARTP/structural-analogue screening also showed stable yield improvement [49]. Earlier chemical/physical mutagenesis studies and reviews of mutagenesis technologies for food microorganisms indicate that random breeding remains an important route acceptable to the food industry [50,51].

Historically, MK-7 production by natto strains has been significantly improved through culture selection and analogue resistance [52,53]. Therefore, co-production engineering should not pursue only a single extreme MK-7 value, but should instead be designed around shared constraints: for example, reducing overflow, improving peptide release, enhancing secretion while limiting nonspecific proteolysis, increasing membrane capacity, and maintaining the food phenotype. For any genetic modification, NK, MK-7, γ-PGA/biofilm formation, sporulation, sensory properties, and safety indicators must be measured simultaneously to determine whether it is truly suitable for dual-functional foods.

## 4. Dynamic Fermentation Strategies for Balancing Co-Production

The metabolic trade-offs and regulatory differences between NK and MK-7 ultimately converge into a process-control challenge. Although *Bacillus subtilis* natto can simultaneously produce both products, NK and MK-7 differ in production characteristics, cellular localization, and accumulation behavior. NK is mainly evaluated through extracellular enzymatic activity, whereas MK-7 accumulation is associated with cellular metabolism and membrane-associated processes. Therefore, improving NK-MK-7 co-production requires fermentation strategies capable of balancing different production objectives rather than optimizing a single product independently.

This section discusses the major fermentation parameters affecting co-production performance, evaluates current evidence supporting dynamic fermentation strategies, analyzes their limitations and generalizability, and discusses future optimization approaches based on multi-objective process control.

### 4.1. Key Process Parameters: Mechanistic Basis

Fermentation performance is determined by the combined effects of carbon sources, nitrogen sources, minerals, inoculum conditions, and oxygen transfer. Response-surface methodology has been widely applied to identify favorable fermentation conditions; however, optimized parameters obtained from individual strain and substrate systems should be interpreted as system-specific results rather than universal fermentation formulas [54].

Among the major fermentation parameters, oxygen-related variables require particular distinction because they describe different levels of the bioprocess. Agitation and aeration are manipulated operating variables; k_L_a and OTR describe oxygen-transfer capacity and performance; DO is a reactor-state variable determined by the balance between oxygen transfer and cellular uptake; and OUR, CER, and ORP can provide complementary information on respiratory and redox physiology. Changes in agitation may therefore alter oxygen transfer together with mixing, shear, gas holdup, carbon dioxide removal, and biofilm structure. For NK–MK-7 co-production, these variables are more appropriately interpreted as an interconnected process-state system than as interchangeable measures of oxygen availability.

For NK production, nutrient composition and feeding strategies directly influence cell growth, protein synthesis, and extracellular enzyme accumulation. Batch and fed-batch studies have shown that maintaining nutrient availability can improve NK production, although the optimal strategy depends on strain characteristics and reactor conditions [55]. Different low-cost substrates, including soybean milk/glucose systems, protein-rich substrates, and glycerol combined with soybean curd residue, have also been investigated to improve NK production efficiency [56,57,58]. An oxygen-enriched fed-batch strategy achieved 18,014 ± 112 IU/mL NK, indicating that oxygen transfer capacity can become an important limitation during high-level extracellular enzyme production [59].

For MK-7 production, medium composition, solid-state fermentation conditions, and downstream recovery approaches strongly influence the apparent yield. Previous studies using medium optimization, solid-state fermentation, and intensified recovery strategies demonstrated that MK-7 production is affected by both biosynthetic capacity and cellular/product distribution characteristics [60,61,62].

For dynamic NK–MK-7 co-production, the practical objective is therefore to coordinate operating conditions with the evolving physiological state of the culture. Early-stage operation can be designed to support biomass formation and extracellular NK production, whereas subsequent operating conditions can be adjusted according to the physiological and metabolic state associated with MK-7 accumulation. A transferable control framework would pair manipulated variables, including agitation, aeration, and feeding, with measured process and physiological responses such as DO, oxygen-transfer characteristics, OUR, CER, ORP, substrate consumption, and product-formation trends. This process-state-based framework provides a mechanistic basis for determining stage transitions and for developing physiology-guided dynamic fermentation strategies.

### 4.2. Experimental Evidence and Rationale for Dynamic Co-Production

Available experimental studies indicate that NK-MK-7 co-production can be achieved through several complementary routes, although the strength and purpose of the evidence differ among fermentation systems. Direct co-production studies have mainly focused on fermentation-condition regulation, redistribution of intracellular resources, and integration of fermentation with downstream recovery, whereas other studies have simultaneously quantified both functional outputs in food matrices or during strain screening. These complementary observations broaden the experimental basis for evaluating co-production beyond a small number of explicitly designed dual-product processes.

Wang et al. established one of the earliest direct co-production systems using soybean curd residue and online dissolved-oxygen regulation [18]. The process produced 2675.73 U/mL NK and 91.25 mg/L MK-7, while the responses of the two products to oxygen availability differed during fermentation. Higher oxygen availability favored biomass formation and NK production, whereas lower dissolved oxygen during the later stage was associated with MK-7 accumulation. Although the optimal oxygen profile is specific to the strain, substrate, and reactor configuration used, this study demonstrated that a single fixed cultivation condition may not maximize both products throughout the entire fermentation process.

A complementary strategy was demonstrated by Huang et al., who reduced 2,3-butanediol overflow metabolism through deletion of *bdhA* [19]. The resulting strain produced 6.5 FU/mL NK and 30.6 mg/L MK-7, accompanied by a 64% reduction in 2,3-butanediol formation. These results support the possibility that decreasing a competing carbon sink can improve both production objectives through broader redistribution of intracellular resources. However, the observed improvements should not be interpreted as direct evidence of precursor competition between the NK and MK-7 pathways, because the intervention affects central metabolism more broadly.

Product localization provides another route for coordinating multi-product fermentation. Yi et al. combined biofilm fermentation with sequential extraction of NK, MK-7, and γ-PGA from a single *B. subtilis* natto fermentation system [17]. The process achieved an NK activity of 2949.3 U/mL and extraction recoveries of 77.1% for NK and 75.4% for MK-7. This approach illustrates that co-production performance depends not only on intracellular synthesis but also on whether the different cellular distributions of the products can be exploited during downstream recovery.

Evidence from food fermentation systems further indicates that the relationship between NK and MK-7 is strongly dependent on the strain–matrix combination. Rocchi et al. compared three *B. subtilis* var. natto strains across different legume substrates and found substantial differences in both MK-7 content and fibrinolytic activity [63]. Strain 92 generally produced relatively high MK-7 levels but showed the lowest NK production among the tested strains. This divergence is important for co-production-oriented strain selection because a strain selected according to a single-product phenotype may not provide the most balanced dual-output performance.

More direct evidence for temporal separation has recently been provided by Maneesha and Subathra Devi [20]. In *B. subtilis* MM26, NK production reached 155.1 ± 1.24 U/mL with a maximum at 72 h, whereas MK-7 reached 67 ± 0.6 mg/L with its maximum at 120 h. The approximately 48 h separation between the two production maxima provides experimental support for asynchronous production kinetics within the same fermentation system. Rather than implying a universal switching time, this observation strengthens the rationale for adjusting cultivation conditions according to the physiological and production state of the culture.

Other dual-output studies extend this concept toward strain selection and food application. Simultaneous screening of fibrinolytic activity and MK-7 production has been used to identify candidate *B. subtilis* strains before subsequent MK-7-oriented engineering [64]. In fermented dairy cream, both functional outputs could also be enhanced under a common fermentation condition, demonstrating that NK-MK-7 dual functionality is not restricted to conventional soybean matrices [65]. More complex sequential food fermentation systems have further retained both NK and MK-7 together with additional metabolites such as γ-PGA and monacolin K [66]. These studies should not be interpreted as direct optimization of NK-MK-7 co-production, but they demonstrate the broader applicability of dual- and multi-output fermentation concepts.

Taken together, the available evidence reveals three major forms of coordination: regulation of the fermentation environment, redistribution of metabolic resources, and exploitation of differences in product localization and production timing. Importantly, the reported systems do not indicate a universal condition that simultaneously maximizes NK and MK-7. Instead, strain-dependent trade-offs, matrix effects, and asynchronous production maxima suggest that dynamic fermentation should be considered a process framework in which environmental conditions are adjusted according to changing physiological and production states. Two-stage fermentation therefore represents one practical implementation of this broader principle rather than a fixed protocol applicable to all strains and fermentation systems. The representative evidence and its current limitations are summarized in Table 2.

### 4.3. Limitations and Unresolved Issues

Despite the expanding experimental evidence summarized above, directly optimized NK-MK-7 co-production systems remain relatively limited, and the available studies differ substantially in strain background, fermentation matrix, cultivation mode, analytical method, and production objective. Direct co-production studies, dual-output food fermentations, and extended multi-product systems therefore provide complementary but not equivalent levels of evidence. This heterogeneity limits direct comparison of reported yields and makes it difficult to define universally applicable operating conditions.

A central unresolved issue is the criterion used to determine when fermentation conditions should be changed. Current dynamic strategies are generally based on cultivation time, dissolved oxygen, agitation, or other externally controlled process variables. However, the observed temporal separation between NK and MK-7 production indicates that a fixed switching time cannot be assumed to apply across strains and fermentation systems [20]. A more transferable strategy would identify stage transitions according to physiological state rather than chronological time. Candidate indicators may include substrate-consumption rate, oxygen uptake and carbon dioxide evolution, redox potential, biomass-specific product formation, and changes associated with the transition from active growth to stationary-phase metabolism.

A second limitation is the lack of real-time or near-real-time measurements for the two target products. NK and MK-7 are generally determined using offline assays, while conventional online variables such as dissolved oxygen, pH, and agitation primarily reflect the fermentation environment rather than product formation itself. Consequently, changes in process variables cannot be directly interpreted as changes in NK secretion or MK-7 biosynthetic flux. Future dynamic control will therefore require either rapid analytical methods or validated soft sensors that integrate conventional process signals with periodically measured product and metabolic data.

The complexity is further increased in solid-state and food matrix fermentation. Temperature, moisture, oxygen transfer, nutrient accessibility, and biofilm development can vary spatially within the substrate, while the matrix itself can influence both metabolite production and the measurable activity of extracellular enzymes. The strain–matrix differences observed across legume substrates [63] illustrate that fermentation performance cannot be predicted from strain characteristics alone. Therefore, optimization of NK-MK-7 co-production must consider the interaction among strain, substrate, fermentation environment, and downstream recovery rather than treating these factors independently.

Finally, improvements in product yield must be interpreted together with food-quality and process-performance requirements. Conditions that enhance one functional component may alter γ-PGA formation, texture, flavor, biomass characteristics, or downstream recovery. Thus, the remaining challenge is not simply to identify conditions that increase both NK and MK-7, but to establish robust operating windows in which dual-product performance can be maintained together with analytical reliability, product quality, and process stability.

### 4.4. Multi-Objective Optimization and Process Trade-Offs

NK-MK-7 co-production represents an inherently multi-objective optimization problem because improving one production objective may influence other physiological or process-related parameters. Therefore, fermentation optimization should not focus only on maximizing NK activity or MK-7 concentration, but should consider a broader set of objectives, including volumetric productivity, fermentation duration, raw-material utilization, energy consumption, product stability, and food-quality characteristics.

Available co-production evidence further demonstrates that the optimal conditions for different production objectives do not always coincide. This divergence can occur at several levels. In strain–matrix comparisons, strains exhibiting relatively high MK-7 production did not necessarily show correspondingly high NK production [63]. Temporal differences have also been observed, with NK and MK-7 reaching their production maxima at 72 and 120 h, respectively, in *B. subtilis* MM26 [20]. Similarly, phase-dependent oxygen regulation affected the two products differently in the soybean-curd-residue co-production system [18]. Nevertheless, such trade-offs are not necessarily absolute. In the bdhA-deletion study, reducing 2,3-butanediol formation was accompanied by increased production of both NK and MK-7 [19], indicating that the relationship between the two products can shift with the metabolic background and fermentation context rather than remaining strictly competitive. Therefore, the objective of co-production optimization should not be to maximize either product independently, but to identify an operating range that provides an appropriate balance between the two outputs and other process requirements.

This interpretation is also consistent with the broader literature on the independent optimization of the two products. Previous studies on MK-7 enhancement have demonstrated that strain selection, medium composition, redox regulation, and membrane-associated engineering approaches can substantially influence production performance [67]. Meanwhile, studies focusing on NK production have shown that substrate composition and fermentation conditions also affect enzyme activity and product quality characteristics [68]. These findings indicate that NK-MK-7 co-production requires coordinated optimization rather than independent improvement of individual products.

At the experimental level, statistical optimization approaches can be used to identify major influencing factors and establish relationships among fermentation parameters, NK and MK-7 responses, and quality indicators. For co-production studies, the two target products should therefore be evaluated simultaneously rather than optimized sequentially as independent endpoints. However, optimization results should be interpreted within the specific strain and fermentation system because interactions among biological and engineering variables may limit direct transferability.

For industrial applications, robustness should also be considered as an important optimization objective. A process that achieves slightly lower maximum productivity but maintains stable performance under variations in raw materials, temperature, oxygen transfer, and batch conditions may be more valuable than a process achieving a higher yield only under tightly controlled laboratory conditions. Therefore, future NK-MK-7 fermentation systems should move from single-parameter optimization toward multi-objective decision frameworks that balance productivity, stability, quality, and economic feasibility. For food applications, co-production performance should also be evaluated at the final-product level. Processing and storage may affect NK activity and MK-7 stability, while the food matrix can influence sensory quality and the nutritional availability of the two products. Although dual-functional fermentation has been demonstrated in food matrices with acceptable sensory properties [63], consumer acceptance and the retention of NK and MK-7 functionality during storage remain important considerations for future product development.

### 4.5. Scale-Up Challenges: From Laboratory to Industrial Production

Although dynamic fermentation strategies provide a potential framework for improving NK–MK-7 co-production, scale-up remains challenging because oxygen transfer, mixing, heat removal, substrate distribution, and process monitoring change with reactor scale. Scale-up should therefore be evaluated using transferable engineering variables such as the volumetric oxygen-transfer coefficient (kLa), power input per unit volume (P/V), impeller tip velocity (Vtip), and mixing time rather than by reproducing laboratory agitation speeds or bulk DO set points. In a recent *B. subtilis* natto biofilm study, scale-up from 2 to 30 L was compared using kLa, P/V, and Vtip. Constant-kLa scale-up produced 21.0 ± 1.0 mg/L MK-7, close to 20.6 ± 1.0 mg/L at the 2 L scale, while P/V and Vtip varied rather than being simultaneously conserved [69]. Thus, maintaining one scale-up criterion does not necessarily preserve the other hydrodynamic variables. NK production is likewise sensitive to oxygen-transfer conditions: in a 5 L B. subtilis fermentation operated at an air supply of 1.0 vvm, increasing the agitation speed from 450 to 650 rpm increased NK activity from 4859 ± 142 to 12,294 ± 226 IU/mL, and pure-oxygen supplementation further increased it to 15,013 ± 550 IU/mL [59]. These examples support the use of product- and reactor-specific oxygen-transfer and mixing criteria during scale-up, but they do not justify a universal numerical kLa, P/V, or Vtip target for NK–MK-7 co-production.

For solid-state fermentation systems, scale-up challenges are more complex because temperature, moisture, and oxygen gradients may develop within the fermentation matrix. Although intermittent aeration and mechanical mixing can improve environmental uniformity, excessive disturbance may affect surface growth, biofilm formation, γ-PGA accumulation, and traditional natto characteristics. Therefore, engineering strategies should balance environmental control with preservation of desirable food fermentation properties.

Downstream processing also influences the feasibility of industrial co-production. Because NK mainly exists in extracellular fractions whereas MK-7 is associated with cells and membrane components, their different distributions provide opportunities for sequential recovery and product separation. However, extraction conditions, including temperature, solvent exposure, oxidation, and processing time, may affect product stability and should be optimized together with fermentation conditions.

Furthermore, industrial evaluation should not rely solely on reported NK activity or MK-7 concentration. Differences in analytical methods, substrate costs, energy consumption, recovery efficiency, and batch consistency may significantly influence practical feasibility. Therefore, future scale-up studies should integrate fermentation performance with techno-economic evaluation and standardized analytical approaches to determine whether laboratory-scale achievements can be translated into reliable food-production systems.

## 5. From Laboratory to Industry: Analytical Standardization, Safety Evaluation, and Research Roadmap

The transition of NK-MK-7 co-production from laboratory research toward practical food applications requires more than improving fermentation performance. Standardized analytical methods, reliable safety evaluation, regulatory compatibility, and industrial process validation are essential for determining whether laboratory-scale achievements can be translated into stable and commercially feasible products.

Although previous studies have demonstrated the feasibility of enhancing NK and MK-7 production through fermentation optimization, metabolic regulation, and process control, differences in analytical methods, strain characteristics, and evaluation criteria remain major barriers for comparison among studies. Therefore, future development requires coordinated efforts in analytical standardization, safety assessment, and industrial implementation.

### 5.1. Challenges and Solutions for Standardizing Analytical Methods

Reliable analytical methods are fundamental for evaluating NK-MK-7 co-production performance. However, current studies frequently apply different analytical strategies, making direct comparison of reported production levels difficult.

For NK, activity evaluation remains challenging because different studies use different measurement systems, including fibrin degradation assays, fibrinolytic units, and enzyme activity calculations. Variations in substrate composition, reaction conditions, incubation time, and calibration standards may introduce substantial differences among reported values. Therefore, NK evaluation should clearly distinguish enzyme quantity, enzymatic activity, and functional performance.

For industrial quality control, future studies should report detailed assay conditions, reference standards, detection limits, and reproducibility parameters. In addition, specific activity may provide more meaningful information than total activity alone because fermentation products may contain different protein compositions and extracellular components.

For MK-7, analytical standardization requires particular attention to molecular forms and isomer composition. Previous studies have developed HPLC-based methods for MK-7 quantification and demonstrated the importance of separating all-trans and cis-MK-7 forms during analysis [70,71]. Since different MK-7 configurations may exhibit different biological characteristics, analytical methods should clearly define whether reported values represent total MK-7 or biologically relevant all-trans MK-7 content [72].

Furthermore, fermentation conditions and storage processes may influence MK-7 stability and isomer distribution. Studies have shown that environmental conditions during production and storage can affect MK-7 composition, highlighting the importance of standardized sample preparation and analytical procedures [73]. Advanced analytical approaches have also been developed for high-purity MK-7 characterization, providing potential references for future industrial quality evaluation [74].

Therefore, establishing harmonized analytical protocols is essential for comparing NK-MK-7 production systems and evaluating improvements in fermentation performance. Without methodological consistency, differences among reported yields may reflect analytical variation rather than true biological improvement.

### 5.2. Safety Considerations and Selected International Regulatory Examples

The industrial application of NK-MK-7 co-production requires comprehensive safety evaluation integrating microbial characteristics, product properties, and evidence-based health assessment.

For NK-containing products, current human studies have investigated potential cardiovascular-related effects. A systematic review and meta-analysis summarized randomized clinical studies and suggested that NK supplementation may influence several cardiovascular risk-related indicators; however, differences in dosage, study population, and experimental design limit definitive conclusions [75]. Several clinical trials reported changes in blood pressure-related parameters or coagulation indicators after NK supplementation [76,77,78], whereas longer-term investigations did not demonstrate consistent improvements in all evaluated cardiovascular endpoints [79]. Toxicological assessment of nattokinase derived from *B. subtilis* var. natto has also provided complementary evidence for its safety evaluation [80]. Therefore, NK-containing foods should avoid unsupported therapeutic claims, and functional descriptions should remain consistent with available evidence.

For MK-7-containing products, safety evaluation should consider both nutritional benefits and potential interactions with specific populations. Vitamin K intake may influence anticoagulant therapy, and studies have shown that MK-7 supplementation can affect vitamin K status in individuals receiving vitamin K antagonist treatment [81,82]. Meanwhile, available safety evaluations have generally supported the use of MK-7 under appropriate nutritional conditions, based on animal and human studies [83,84].

For products containing live microbial strains, safety assessment should be performed at the strain level. Important considerations include microbial identity, genomic stability, antimicrobial resistance characteristics, and potential safety risks. Although nattokinase-producing and MK-7-producing *Bacillus subtilis* strains have a long history of food application, industrial production requires systematic characterization of individual production strains.

EFSA Qualified Presumption of Safety (QPS) provides a framework for evaluating microbial groups with established safety histories. However, QPS status should be interpreted as a risk-assessment approach rather than automatic approval of every strain or product. Individual strains still require evaluation according to their characteristics and intended application [85].

Regulatory requirements for NK-MK-7 products also depend on product category and target market. In the European Union, nutrition and health claims are regulated under Regulation (EC) No 1924/2006, requiring that claimed physiological effects are supported by appropriate scientific evidence [86]. Products involving genetically modified microorganisms or derived ingredients may additionally require evaluation under specific GMO regulations, such as Regulation (EC) No 1829/2003 [87].

Regulatory approaches outside the European Union are likewise product- and use-specific. In Japan, functional foods may fall under the Foods with Nutrient Function Claims, Foods for Specified Health Uses, or Foods with Function Claims frameworks, with different requirements for scientific substantiation and regulatory review [88]. A specific NK precedent is available under the Foods with Function Claims system: Consumer Affairs Agency records for notification F680 (“Nattokinase Sarasara Tsubu”) identify nattokinase derived from natto bacteria as the functional ingredient [89]. This notification should not be interpreted as a general approval of nattokinase or extrapolated to MK-7. In the United States, GRAS conclusions apply to specific substances and intended uses; FDA issued a “no questions” letter for MK-7 under GRN 887 [90], whereas no corresponding nattokinase notice was identified in the public FDA GRAS Notice Inventory [91]. This absence should not be interpreted as FDA disapproval, and dietary supplements themselves are generally not subject to FDA premarket approval [92]. In China, fermented vitamin K2 has been authorized as a nutrient fortifier for specified formulated milk-powder categories [93]. For NK, a narrower provincial precedent is available: Hubei Province’s DBS42/020—2026 standard regulates natto powder as a food ingredient and explicitly defines nattokinase and its activity specifications; however, this provincial standard should not be interpreted as nationwide authorization of purified nattokinase [94]. These differences indicate that the regulatory feasibility of an NK–MK-7 co-product must be evaluated according to its final form, production strain, intended use, and target market.

Therefore, future industrial development should consider regulatory requirements as an integral component of product design rather than a final approval step. Safety evaluation, analytical standardization, and evidence-based health communication should be integrated throughout the development process.

### 5.3. Bridging Knowledge Gaps: A Phased Research Roadmap

**Figure 3:**
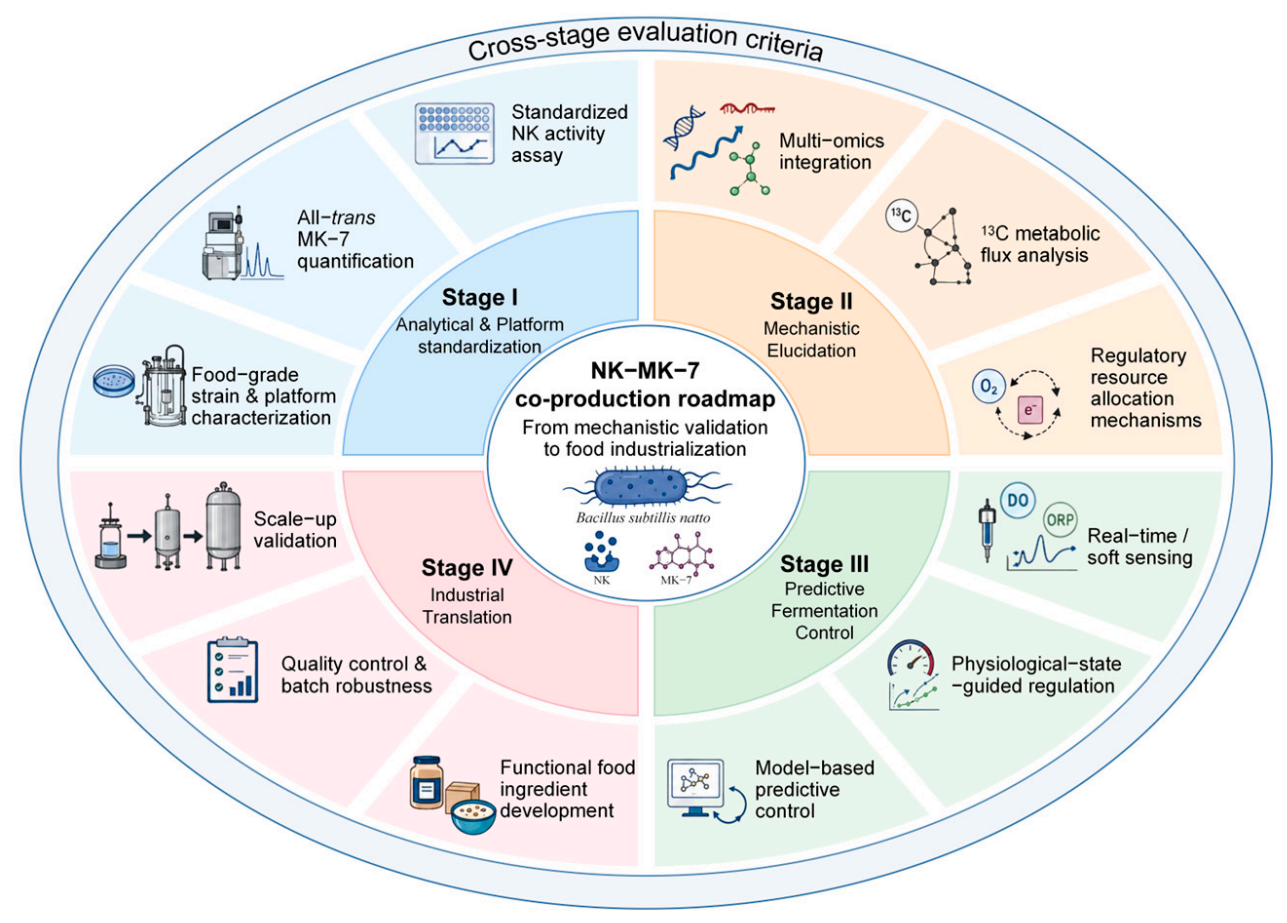
Proposed roadmap for NK-MK-7 co-production from mechanistic validation to food industrialization. The roadmap comprises four progressive stages: (I) analytical and platform standardization, including food-grade strain and production platform characterization, all-trans MK-7 quantification, and standardized NK activity assays; (II) mechanistic elucidation through multi-omics integration, ^13^C metabolic flux analysis, and clarification of regulatory and resource allocation mechanisms; (III) predictive fermentation control based on real-time or soft sensing, physiological-state-guided regulation, and model-based predictive control; and (IV) industrial translation through scale-up validation, quality control and batch robustness, and functional food or ingredient development. Cross-stage evaluation should consider analytical comparability, safety, product quality, process robustness, and economic feasibility.

Although NK-MK-7 co-production has achieved considerable progress through fermentation optimization, metabolic regulation, and process engineering strategies, several knowledge gaps remain before laboratory-scale findings can be translated into reliable food industrial applications. Future development requires a gradual transition from empirical optimization toward standardized evaluation, mechanistic understanding, and predictive process control. The proposed roadmap for NK-MK-7 co-production, from mechanistic validation to food industrialization, is illustrated in Figure 3.

A fundamental step toward industrial translation is the establishment of analytical comparability and standardized production platforms. Current studies often employ different approaches for evaluating NK activity, MK-7 concentration, and fermentation performance, which limits direct comparison among reported results. Therefore, future research should further harmonize analytical procedures, particularly regarding NK functional activity assessment and all-trans MK-7 quantification. Meanwhile, representative food-grade production strains should undergo systematic characterization, including strain identification, safety evaluation, fermentation phenotype analysis, and process-performance assessment. Standardized fermentation platforms integrating environmental monitoring and process-data acquisition will provide reliable foundations for subsequent optimization and industrial evaluation.

Following the establishment of standardized platforms, future studies should focus on mechanistic validation and dynamic process control. Although current evidence suggests that oxygen regulation, carbon-flow redistribution, and spatial organization may influence NK-MK-7 co-production, the quantitative relationships among environmental conditions, intracellular resource allocation, and product formation remain insufficiently understood. Therefore, further investigations should integrate metabolic analysis, physiological monitoring, and fermentation engineering approaches to clarify key regulatory factors and develop adaptive control strategies. Rather than relying on empirical switching points, future fermentation systems should aim to achieve physiological-state-guided regulation based on measurable changes in microbial metabolism and product accumulation.

The final objective is the development of robust and predictive production systems capable of maintaining stable performance under variable manufacturing conditions. Future strategies should integrate strain characteristics, substrate variability, fermentation parameters, downstream processing, and product-quality evaluation. Data-driven optimization and predictive control approaches may provide potential solutions for improving batch consistency and reducing process uncertainty; however, their application should remain compatible with food safety requirements, regulatory acceptance, economic feasibility, and consumer expectations.

Overall, successful NK-MK-7 co-production should not be evaluated solely by maximum production yield. Instead, industrial feasibility should be assessed through a comprehensive framework integrating analytical reliability, safety evidence, process robustness, economic performance, and product quality.

## 6. Conclusions

The co-production of nattokinase (NK) and menaquinone-7 (MK-7) by *Bacillus subtilis* natto represents a promising strategy for developing multifunctional fermented foods and multi-product microbial fermentation systems. However, coordinated production of these two metabolites is constrained by their distinct biosynthetic characteristics, cellular localization, and physiological requirements. Current evidence most clearly demonstrates differences in product localization and, in specific fermentation systems, production timing and responses to process conditions; these observations represent process-level constraints rather than proof of direct metabolic competition. Associations involving energy and redox regulation are supported by physiological and omics evidence but remain system-dependent, whereas direct precursor-level competition—particularly involving chorismate allocation—has not been quantitatively demonstrated and should remain a mechanistic hypothesis requiring further validation.

Multi-omics studies have substantially improved understanding of how fermentation substrates, oxygen availability, and cellular regulatory networks influence NK and MK-7 production. Nevertheless, most available studies remain based on condition-dependent associations rather than direct quantitative relationships between gene regulation, metabolic flux redistribution, and product accumulation. Therefore, future research should integrate transcriptomics, metabolomics, ^13^C metabolic flux analysis, and genome-scale modeling to establish a quantitative framework describing carbon allocation, energy utilization, and redox balance during co-production.

Dynamic fermentation strategies, particularly two-stage fermentation approaches, provide a useful conceptual framework for coordinating the different physiological requirements of NK and MK-7. However, current evidence is mainly derived from specific strains, substrates, and reactor systems, and the optimal transition point between production phases remains unclear. Future process development should move beyond empirical stage switching toward physiology-based control strategies supported by real-time monitoring of metabolic states, online sensing technologies, and multi-objective optimization.

For industrial translation, maximizing product yield alone is insufficient. Reliable analytical standardization, including comparable NK activity assays and MK-7 isomer quantification, together with strain safety evaluation, solid-state fermentation scale-up, and economic assessment, will be essential for transforming laboratory demonstrations into food-grade production systems. Overall, NK-MK-7 co-production is transitioning from empirical optimization toward mechanism-guided design. Establishing quantitative metabolic models, predictive fermentation control, and standardized quality frameworks will provide a foundation for future development of dual-functional fermented foods and broader multi-product microbial bioprocesses.

## Figures and Tables

**Table 1:** Comparison of the major metabolic and process requirements for NK and MK-7 production.

Dimension	NK	MK-7	Implication for Co-Production
Product property/localization	Secreted serine protease; extracellular activity is the main endpoint	Lipid-soluble respiratory quinone; mainly associated with the cell membrane/solid phase	Downstream fractionation is possible, but the optimal harvest times differ
Key material requirements	Amino acids/peptides, protein translation, and secretion capacity	Chorismate-DHNA and MEP isoprenoid precursors	Chorismate competition is currently a mechanistic hypothesis and requires flux validation
Energy/reducing power	ATP, proton motive force, and high-level protein synthesis	MEP reducing power, respiratory electron transfer, and membrane homeostasis	OUR, CER, ORP, and related physiological signals are candidate indicators for future dynamic monitoring.
Oxygen/redox trend	Adequate oxygen transfer generally supports growth and secretion	Can be enhanced under high oxygen or structured/dynamic states depending on the system	There is no universal “low-oxygen optimum for MK-7”
Temporal preference	High secretion is common in late logarithmic/early stationary phase; later stages require protection against inactivation	May accumulate with cell maturation, membrane state, and biofilm formation	Supports stage control triggered by physiological state
Major limiting factors	Expression, Sec capacity, extracellular degradation/adsorption, and assay units	Precursors, MenD/MenA, membrane capacity, extraction, and isomers	Optimization must incorporate both sensory quality and analytical comparability

Note: This table represents an author synthesis based on the mechanistic and experimental evidence discussed in Section 2.1, Section 2.2 and Section 2.3. Not all listed relationships represent directly demonstrated metabolic competition; some should be interpreted as process-level constraints or mechanistic hypotheses requiring further quantitative validation.

**Table 2:** Representative experimental evidence for simultaneous NK and MK-7 production and co-production optimization.

Study	Strain/System	Evidence Type and Strategy	NK Result	MK-7 Result	Main Implication for Co-Production
Yi et al., 2025 [17]	*B. subtilis natto* CICC 25137; soybean-meal hydrolysate	Direct multi-product co-production; biofilm fermentation + sequential extraction	2949.3 U/mL; 77.1% extraction recovery	75.4% extraction recovery	Integrates NK, MK-7, and γ-PGA production with downstream fractionation, showing that product localization can be exploited for multi-product recovery; analytical units should be considered carefully in cross-study comparisons.
Wang et al., 2018 [18]	*B. subtilis*; soybean-curd-residue system	Direct co-production; medium optimization + online DO control	2675.73 U/mL	91.25 mg/L	Increasing oxygen availability favored growth and NK production, whereas lower DO promoted late-stage MK-7 accumulation, supporting phase-dependent regulation; the optimal profile remains system-specific.
Huang et al., 2024 [19]	*B. subtilis* 168 Δ*bdhA*	Direct co-production; reduction of 2,3-butanediol overflow + RNA-seq	6.5 FU/mL	30.6 mg/L	2,3-Butanediol was reduced by 64%, supporting redistribution of carbon from an overflow sink toward both products; this does not by itself demonstrate direct precursor competition between NK and MK-7.
Maneesha and Subathra Devi, 2026 [20]	*B. subtilis* MM26 isolated from fermented grape wine	Direct co-production; strain screening + time-course fermentation	155.1 ± 1.24 U/mL; maximum at 72 h	67 ± 0.6 mg/L; maximum at 120 h	The different production maxima provide direct evidence of temporal mismatch between NK and MK-7 formation and strongly support stage-specific fermentation control.
Rocchi et al., 2024 [63]	Three *B. subtilis* var. natto strains; six legume substrates	Dual-output food fermentation; strain × substrate comparison	5–350 NK units/g across fermented samples; 347 units/g in soybean fermented with strain 2.8	78.9–2002 μg/100 g; highest value in chickpea fermented with strain 92	Strain 92 generally produced the most MK-7 but showed the lowest NK production, providing evidence that strain selection based on a single product may not identify the best co-production phenotype.
Li et al., 2025 [64]	*B. subtilis* isolates from commercial natto starters; strain L-5 selected	Dual-output strain screening prior to MK-7 engineering	646.49 ± 3.2 IU/mL fibrinolytic activity in L-5 (urokinase-equivalent assay)	75.18 ± 0.42 mg/L in L-5	Demonstrates the value of screening both fibrinolytic activity and MK-7 before strain engineering; subsequent engineering focused primarily on MK-7 and therefore does not constitute direct co-production optimization.
Moharampour et al., 2026 [65]	*B. subtilis natto*; fermented dairy cream	Dual-output food fermentation; temperature × fermentation-time optimization	32.87 ± 1.14 FU/g at 30 °C for 24 h	44.61 ± 0.59 ng/g at 30 °C for 24 h	Both functional outputs were favored under the same tested condition, demonstrating that dual-functional fermentation can be transferred from traditional legume matrices to a dairy food matrix while retaining acceptable sensory properties.
Uthai et al., 2026 [66]	Natto–angkak two-step fermentation system	Extended multi-product food fermentation; sequential fermentation	1211 U/g on day 25	12.88 mg/kg on day 25	Demonstrates retention of both NK and MK-7 within a more complex multi-product food fermentation producing γ-PGA and monacolin K; it should be regarded as process-level extension rather than single-strain NK–MK-7 co-optimization.

Note: NK activity is reported using study-specific activity units (e.g., U, FU, IU, or units per mass), whereas MK-7 is reported using concentration- or matrix-based units (e.g., mg/L, μg/100 g, ng/g, or mg/kg). Values should therefore not be interpreted as directly comparable across studies because assay principles, substrates, fermentation matrices, extraction procedures, and normalization methods differ. See Section 5.1 for discussion of analytical standardization.

## Data Availability

No new data were created or analyzed in this study. Data sharing is not applicable to this article.

## Abbreviations

The following abbreviations are used in this manuscript:

^13^C-MFA	^13^C metabolic flux analysis
ARTP	Atmospheric and room temperature plasma
ATP	Adenosine triphosphate
CER	Carbon dioxide evolution rate
DHNA	1,4-dihydroxy-2-naphthoic acid
DMAPP	Dimethylallyl diphosphate
DO	Dissolved oxygen
EFSA	European Food Safety Authority
FU	Fibrinolytic unit
γ-PGA	Poly-γ-glutamic acid
GMO	Genetically modified organism
HPLC	High-performance liquid chromatography
IPP	Isopentenyl diphosphate
IU	International unit
MEP	2-C-methyl-D-erythritol 4-phosphate
MK-7	Menaquinone-7
NAD(P)H	Reduced nicotinamide adenine dinucleotide (phosphate)
NK	Nattokinase
ORP	Oxidation–reduction potential
OUR	Oxygen uptake rate
RNA-seq	RNA sequencing
ROS	Reactive oxygen species

## References

[1] Kubo Y., Rooney A.P., Tsukakoshi Y., Nakagawa R., Hasegawa H., Kimura K. (2011). Phylogenetic Analysis of *Bacillus subtilis* Strains Applicable to Natto (Fermented Soybean) Production. Appl. Environ. Microbiol..

[2] Nishito Y., Osana Y., Hachiya T., Popendorf K., Toyoda A., Fujiyama A., Itaya M., Sakakibara Y. (2010). Whole genome assembly of a natto production strain *Bacillus subtilis* natto from very short read data. BMC Genom..

[3] Kamada M., Hase S., Fujii K., Miyake M., Sato K., Kimura K., Sakakibara Y. (2015). Whole-Genome Sequencing and Comparative Genome Analysis of *Bacillus subtilis* Strains Isolated from Non-Salted Fermented Soybean Foods. PLoS ONE.

[4] Seki K., Nagano Y. (2025). Conserved accessory genes link a phylogenetically distinct *Bacillus subtilis* strain from Indian bekang to the Japanese natto clade. Sci. Rep..

[5] Sumi H., Hamada H., Tsushima H., Mihara H., Muraki H. (1987). A novel fibrinolytic enzyme (nattokinase) in the vegetable cheese Natto; a typical and popular soybean food in the Japanese diet. Experientia.

[6] Fujita M., Nomura K., Hong K., Ito Y., Asada A., Nishimuro S. (1993). Purification and characterization of a strong fibrinolytic enzyme (nattokinase) in the vegetable cheese natto, a popular soybean fermented food in Japan. Biochem. Biophys. Res. Commun..

[7] Dabbagh F., Negahdaripour M., Berenjian A., Behfar A., Mohammadi F., Zamani M., Irajie C., Ghasemi Y. (2014). Nattokinase: Production and application. Appl. Microbiol. Biotechnol..

[8] Cai D., Zhu C., Chen S. (2017). Microbial production of nattokinase: Current progress, challenge and prospect. World J. Microbiol. Biotechnol..

[9] Sheng Y., Yang J., Wang C., Sun X., Yan L. (2023). Microbial nattokinase: From synthesis to potential application. Food Funct..

[10] Berenjian A., Mahanama R., Kavanagh J., Dehghani F. (2015). Vitamin K series: Current status and future prospects. Crit. Rev. Biotechnol..

[11] Tarvainen M., Fabritius M., Yang B. (2019). Determination of vitamin K composition of fermented food. Food Chem..

[12] Sato T., Schurgers L.J., Uenishi K. (2012). Comparison of menaquinone-4 and menaquinone-7 bioavailability in healthy women. Nutr. J..

[13] Theuwissen E., Cranenburg E.C., Knapen M.H., Magdeleyns E.J., Teunissen K.J., Schurgers L.J., Smit E., Vermeer C. (2012). Low-dose menaquinone-7 supplementation improved extra-hepatic vitamin K status, but had no effect on thrombin generation in healthy subjects. Br. J. Nutr..

[14] Knapen M.H.J., Drummen N.E., Smit E., Vermeer C., Theuwissen E. (2013). Three-year low-dose menaquinone-7 supplementation helps decrease bone loss in healthy postmenopausal women. Osteoporos. Int..

[15] Knapen M.H.J., Braam L.A.J.L.M., Drummen N.E., Bekers O., Hoeks A.P.G., Vermeer C. (2015). Menaquinone-7 supplementation improves arterial stiffness in healthy postmenopausal women: A double-blind randomised clinical trial. Thromb. Haemost..

[16] Vossen L.M., de Leeuw P.W., Schurgers L.J., Heuts S., Adriaans B.P., de Haan C., van Varik B.J., Kroon A.A. (2026). Two Years of Menaquinone-7 Supplementation and Coronary Artery Calcification: A Randomized Clinical Trial. JAMA Cardiol..

[17] Yi Y., Chen M., Coldea T.E., Zhao H. (2025). A novel integrated bioprocessing strategy for co-production and sequential extraction of menaquinone-7, nattokinase, and γ-polyglutamic acid from *Bacillus subtilis* natto. Food Biosci..

[18] Wang H., Sun X., Wang L., Wu H., Zhao G., Liu H., Wang P., Zheng Z. (2018). Coproduction of menaquinone-7 and nattokinase by *Bacillus subtilis* using soybean curd residue as a renewable substrate combined with a dissolved oxygen control strategy. Ann. Microbiol..

[19] Huang X., Gao X., Huang J., Luo Y., Tao W., Guo M., Liu Y., Wu J., Chen Y., Liu Y. (2024). Study on the effects of *bdhA* knockout on coproduction of menaquinone-7 and nattokinase by *Bacillus subtilis* based on RNA-Seq analysis. Process Biochem..

[20] Maneesha M., Subathra Devi C. (2026). Co-production of nattokinase and menaquinone-7 from *Bacillus subtilis* MM26: A strain isolated from fermented grape wine. Biocatal. Agric. Biotechnol..

[21] Lin Y.-C., Chen E.H.-L., Chen R.P.-Y., Dunny G.M., Hu W.-S., Lee K.-T. (2021). Probiotic *Bacillus* Affects *Enterococcus faecalis* Antibiotic Resistance Transfer by Interfering with Pheromone Signaling Cascades. Appl. Environ. Microbiol..

[22] Tjalsma H., Bolhuis A., Jongbloed J.D.H., Bron S., van Dijl J.M. (2000). Signal Peptide-Dependent Protein Transport in *Bacillus subtilis*: A Genome-Based Survey of the Secretome. Microbiol. Mol. Biol. Rev..

[23] Wang C., Du M., Zheng D., Kong F., Zu G., Feng Y. (2009). Purification and Characterization of Nattokinase from *Bacillus subtilis* Natto B-12. J. Agric. Food Chem..

[24] Ogura M., Shimane K., Asai K., Ogasawara N., Tanaka T. (2003). Binding of response regulator DegU to the aprE promoter is inhibited by RapG, which is counteracted by extracellular PhrG in *Bacillus subtilis*. Mol. Microbiol..

[25] Barbieri G., Albertini A.M., Ferrari E., Sonenshein A.L., Belitsky B.R. (2016). Interplay of CodY and ScoC in the Regulation of Major Extracellular Protease Genes of *Bacillus subtilis*. J. Bacteriol..

[26] Spacapan M., Danevčič T., Mandic-Mulec I. (2018). ComX-Induced Exoproteases Degrade ComX in *Bacillus subtilis* PS-216. Front. Microbiol..

[27] Hata M., Ogura M., Tanaka T. (2001). Involvement of stringent factor RelA in expression of the alkaline protease gene aprE in *Bacillus subtilis*. J. Bacteriol..

[28] Derouiche A., Shi L., Bidnenko V., Ventroux M., Pigonneau N., Franz-Wachtel M., Kalantari A., Nessler S., Noirot-Gros M.-F., Mijakovic I. (2015). *Bacillus subtilis* SalA is a phosphorylation-dependent transcription regulator that represses scoC and activates the production of the exoprotease AprE. Mol. Microbiol..

[29] Driscoll J.R., Taber H.W. (1992). Sequence organization and regulation of the *Bacillus subtilis* menBE operon. J. Bacteriol..

[30] Rowland B., Hill K., Miller P., Driscoll J., Taber H. (1995). Structural organization of a *Bacillus subtilis* operon encoding menaquinone biosynthetic enzymes. Gene.

[31] Dawson A., Chen M., Fyfe P.K., Guo Z., Hunter W.N. (2010). Structure and reactivity of *Bacillus subtilis* MenD catalyzing the first committed step in menaquinone biosynthesis. J. Mol. Biol..

[32] Ma Y., McClure D.D., Somerville M.V., Proschogo N.W., Dehghani F., Kavanagh J.M., Coleman N.V. (2019). Metabolic Engineering of the MEP Pathway for Enhanced Menaquinone-7 Production in *Bacillus subtilis*. ACS Synth. Biol..

[33] Yang S., Cao Y., Sun L., Li C., Lin X., Cai Z., Zhang G., Song H. (2019). Modular Pathway Engineering of *Bacillus subtilis* To Promote De Novo Biosynthesis of Menaquinone-7. ACS Synth. Biol..

[34] Cui S., Xia H., Chen T., Gu Y., Lv X., Liu Y., Li J., Du G., Liu L. (2020). Cell Membrane and Electron Transfer Engineering for Improved Synthesis of Menaquinone-7 in *Bacillus subtilis*. iScience.

[35] Huang W., Li J., Zhu Q., Lv J., Zhu R., Pu C., Zhao H., Fu G., Zhang D. (2024). Increasing Vitamin K2 Synthesis in *Bacillus subtilis* by Controlling the Expression of MenD and Stabilizing MenA. J. Agric. Food Chem..

[36] Berenjian A., Chan N.L.-C., Mahanama R., Talbot A., Regtop H., Kavanagh J., Dehghani F. (2013). Effect of Biofilm Formation by *Bacillus subtilis* natto on Menaquinone-7 Biosynthesis. Mol. Biotechnol..

[37] Wang H., Liu H., Wang L., Zhao G., Tang H., Sun X., Ni W., Yang Q., Wang P., Zheng Z. (2019). Improvement of menaquinone-7 production by *Bacillus subtilis* natto in a novel residue-free medium by increasing the redox potential. Appl. Microbiol. Biotechnol..

[38] Peng C., Zhu S., Lu J., Hu X., Ren L. (2020). Transcriptomic analysis of gene expression of menaquinone-7 in *Bacillus subtilis* natto toward different oxygen supply. Food Res. Int..

[39] Stanley N.R., Lazazzera B.A. (2005). Defining the genetic differences between wild and domestic strains of *Bacillus subtilis* that affect poly-γ-DL-glutamic acid production and biofilm formation. Mol. Microbiol..

[40] Hong L.T.T., Hachiya T., Hase S., Shiwa Y., Yoshikawa H., Sakakibara Y., Nguyen S.L.T., Kimura K. (2019). Poly-γ-glutamic acid production in *Bacillus subtilis* natto in the absence of DegQ: Gain-of-function mutation in yabJ. J. Biosci. Bioeng..

[41] Vlamakis H., Chai Y., Beauregard P., Losick R., Kolter R. (2013). Sticking together: Building a biofilm the *Bacillus subtilis* way. Nat. Rev. Microbiol..

[42] Cui S., Lv X., Wu Y., Li J., Du G., Ledesma-Amaro R., Liu L. (2019). Engineering a Bifunctional Phr60-Rap60-Spo0A Quorum-Sensing Molecular Switch for Dynamic Fine-Tuning of Menaquinone-7 Synthesis in *Bacillus subtilis*. ACS Synth. Biol..

[43] Liu D., Han Z., Hu Z., Yu C., Wang Y., Tong J., Fang X., Yue W., Nie G. (2023). Comparative analysis of the transcriptome of *Bacillus subtilis* natto incubated in different substrates for nattokinase production. Process Biochem..

[44] Li M., Zhang Z., Li S., Tian Z., Ma X. (2021). Study on the mechanism of production of γ-PGA and nattokinase in *Bacillus subtilis* natto based on RNA-seq analysis. Microb. Cell Fact..

[45] Yi Y., Chen M., Coldea T.E., Yang H., Zhao H. (2024). Soy Protein Hydrolysates Induce Menaquinone-7 Biosynthesis by Enhancing the Biofilm Formation of *Bacillus subtilis* natto. Food Microbiol..

[46] Yin X., Wang X., Xu L., Zhao J., Li C., Lin J. (2024). Widely Targeted Metabolomics Revealed the Metabolic Basis of Natto Fermentation. Metabolites.

[47] Sun X., Bi X., Li G., Cui S., Xu X., Liu Y., Li J., Du G., Lv X., Liu L. (2024). Combinatorial metabolic engineering of *Bacillus subtilis* for menaquinone-7 biosynthesis. Biotechnol. Bioeng..

[48] Guo H., Xue S., Dong N., Zhang Y., Dai Y., Chen Y., Zhang S. (2025). Enhancing menaquinone-7 biosynthesis by optimized fermentation strategies and fed-batch cultivation of food-derived *Bacillus subtilis*. J. Sci. Food Agric..

[49] Xu X., Liu Z., Li H., Zhang X., Shi J., Chen J., Duan L., Xu Z. (2021). Mutation and process optimization of *Bacillus* natto ND-1 for vitamin K2 (MK-7) production. Chin. J. Bioprocess Eng..

[50] Song J., Liu H., Wang L., Dai J., Liu Y., Liu H., Zhao G., Wang P., Zheng Z. (2014). Enhanced Production of Vitamin K2 from *Bacillus subtilis* (natto) by Mutation and Optimization of the Fermentation Medium. Braz. Arch. Biol. Technol..

[51] Yu Q., Li Y., Wu B., Hu W., He M., Hu G. (2020). Novel mutagenesis and screening technologies for food microorganisms: Advances and prospects. Appl. Microbiol. Biotechnol..

[52] Sato T., Yamada Y., Ohtani Y., Mitsui N., Murasawa H., Araki S. (2001). Production of menaquinone (vitamin K2)-7 by *Bacillus subtilis*. J. Biosci. Bioeng..

[53] Tsukamoto Y., Kasai M., Kakuda H. (2001). Construction of a *Bacillus subtilis* (natto) with high productivity of vitamin K2 (menaquinone-7) by analog resistance. Biosci. Biotechnol. Biochem..

[54] Deepak V., Kalishwaralal K., Ramkumarpandian S., Venkatesh Babu S., Senthilkumar S.R., Sangiliyandi G. (2008). Optimization of media composition for nattokinase production by *Bacillus subtilis* using response surface methodology. Bioresour. Technol..

[55] Cho Y.-H., Song J.Y., Kim K.M., Kim M.K., Lee I.Y., Kim S.B., Kim H.S., Han N.S., Lee B.H., Kim B.S. (2010). Production of nattokinase by batch and fed-batch culture of *Bacillus subtilis*. New Biotechnol..

[56] Xiao Z., Shen J., Li Y., Wang Z., Zhao Y., Chen Y., Zhao J.-Y. (2022). High and Economical Nattokinase Production with Acetoin as a Useful Byproduct from Soybean Milk and Glucose. Probiotics Antimicrob. Proteins.

[57] Pan S., Chen G., Zeng J., Cao X., Zheng X., Zeng W., Liang Z. (2019). Fibrinolytic enzyme production from low-cost substrates by marine *Bacillus subtilis*: Process optimization and kinetic modeling. Biochem. Eng. J..

[58] Duan Y., Du W., Xu Y., Guo G., Liu Z., Hao N. (2025). Enhanced nattokinase production by *Bacillus subtilis* from glycerol and okara: Optimization of culture medium via response surface methodology. Front. Microbiol..

[59] Zheng J., Sun Y., Liao Y., Qin P., Che R., Zhao J.-Y., Xiao Z. (2025). Dual-purpose *Bacillus subtilis* fermentation: Enhanced nattokinase production via oxygen-enriched fed-batch cultivation and natto starter preparation from harvested biomass. Bioprocess Biosyst. Eng..

[60] Berenjian A., Mahanama R., Talbot A., Biffin R., Regtop H., Valtchev P., Kavanagh J., Dehghani F. (2011). Efficient media for high menaquinone-7 production: Response surface methodology approach. New Biotechnol..

[61] Mahanama R., Berenjian A., Valtchev P., Talbot A., Biffin R., Regtop H., Dehghani F., Kavanagh J.M. (2011). Enhanced Production of Menaquinone 7 via Solid Substrate Fermentation from *Bacillus subtilis*. Int. J. Food Eng..

[62] Berenjian A., Mahanama R., Talbot A., Regtop H., Kavanagh J., Dehghani F. (2014). Designing of an Intensification Process for Biosynthesis and Recovery of Menaquinone-7. Appl. Biochem. Biotechnol..

[63] Rocchi R., Zwinkels J., Kooijman M., Garre A., Smid E.J. (2024). Development of novel natto using legumes produced in Europe. Heliyon.

[64] Li M., Li J., Li Y., Zhang X., Xu J. (2025). Synergistic Optimization of *Bacillus subtilis* for Efficiently Producing Menaquinone-7 (MK-7) by Atmospheric and Room Temperature Plasma (ARTP) Mutagenesis and Metabolic Engineering. Fermentation.

[65] Moharampour H., Azadmard-Damirchi S., Hesari J., Afshar Mogaddam M.R., Farjnia S. (2026). Production of Vitamin K2 Enriched Dairy Cream by *Bacillus subtilis* Natto Fermentation. Food Sci. Nutr..

[66] Uthai N., Wattanakul J., Phanumong P., Kraboun K. (2026). 2-step fermentation of natto angkak: Simultaneous production of MK-7, nattokinase, γ-PGA, and monacolin K, and insights from metabolomics and flavoromics. Appl. Food Res..

[67] Liao C., Ayansola H., Ma Y., Ito K., Guo Y., Zhang B. (2021). Advances in Enhanced Menaquinone-7 Production From *Bacillus subtilis*. Front. Bioeng. Biotechnol..

[68] Li D., Hou L., Hu M., Gao Y., Tian Z., Fan B., Li S., Wang F. (2022). Recent Advances in Nattokinase-Enriched Fermented Soybean Foods: A Review. Foods.

[69] Berenjian A., Mahdinia E., Demirci A. (2025). Scaling up Biofilm Bioreactors for Enhanced Menaquinone-7 Production. Bioprocess Biosyst. Eng..

[70] Celiento A., Giuliani A., Laini M., Longoni M., Rizzi G., Dei Nobili M. (2025). A Novel Fast HPLC-UV Method for Menaquinone-7 Determination in Fermentation Broth. ACS Omega.

[71] Szterk A., Zmysłowski A., Bus K. (2018). Identification of cis/trans isomers of menaquinone-7 in food as exemplified by dietary supplements. Food Chem..

[72] Lal N., Berenjian A. (2020). Cis and trans isomers of vitamin menaquinone-7: Which one is biologically significant?. Appl. Microbiol. Biotechnol..

[73] Lal N., Seifan M., Berenjian A. (2023). Fermentation of Menaquinone-7: The Influence of Environmental Factors and Storage Conditions on the Isomer Profile. Processes.

[74] Jedynak Ł., Jedynak M., Kossykowska M., Zagrodzka J. (2017). A novel method for the determination of the chemical purity and assay of menaquinone-7 and comparison with the USP method. J. Pharm. Biomed. Anal..

[75] Li X., Long J., Gao Q., Pan M., Wang J., Yang F., Zhang Y. (2023). Nattokinase supplementation and cardiovascular risk factors: A systematic review and meta-analysis of randomized controlled trials. Rev. Cardiovasc. Med..

[76] Kim J.Y., Gum S.N., Paik J.K., Lim H.H., Kim K.C., Ogasawara K., Inoue K., Park S., Jang Y., Lee J.H. (2008). Effects of nattokinase on blood pressure: A randomized, controlled trial. Hypertens. Res..

[77] Hsia C.-H., Shen M.-C., Lin J.-S., Wen Y.-K., Hwang K.-L., Cham T.-M., Yang N.-C. (2009). Nattokinase decreases plasma levels of fibrinogen, factor VII, and factor VIII in human subjects. Nutr. Res..

[78] Kurosawa Y., Nirengi S., Homma T., Esaki K., Ohta M., Clark J.F., Hamaoka T. (2015). A single-dose of oral nattokinase potentiates thrombolysis and anti-coagulation profiles. Sci. Rep..

[79] Hodis H.N., Mack W.J., Meiselman H.J., Kalra V., Liebman H., Hwang-Levine J., Dustin L., Kono N., Mert M., Wenby R.B. (2021). Nattokinase atherothrombotic prevention study: A randomized controlled trial. Clin. Hemorheol. Microcirc..

[80] Lampe B.J., English J.C. (2016). Toxicological assessment of nattokinase derived from *Bacillus subtilis var*. natto. Food Chem. Toxicol..

[81] Theuwissen E., Teunissen K.J., Spronk H.M.H., Hamulyák K., Cate H.T., Vermeer C. (2013). Effect of low-dose menaquinone-7 supplements on the stability of oral anticoagulant treatment: Dose-response relationship in healthy volunteers. J. Thromb. Haemost..

[82] National Institutes of Health, Office of Dietary Supplements (2021). Vitamin K: Fact Sheet for Health Professionals. NIH Office of Dietary Supplements. https://ods.od.nih.gov/factsheets/VitaminK-HealthProfessional/.

[83] Ravishankar B., Dound Y.A., Mehta D.S., Ashok B.K., de Souza A., Pan M.-H., Ho C.-T., Badmaev V., Vaidya A.D.B. (2015). Safety assessment of menaquinone-7 for use in human nutrition. J. Food Drug Anal..

[84] Marles R.J., Roe A.L., Oketch-Rabah H.A. (2017). US Pharmacopeial Convention safety evaluation of menaquinone-7, a form of vitamin K. Nutr. Rev..

[85] Allende A., Alvarez-Ordóñez A., Bortolaia V., Bover-Cid S., De Cesare A., Dohmen W., Guillier L., Jacxsens L., Nauta M., Mughini-Gras L. (2025). Update of the list of qualified presumption of safety (QPS) recommended microbiological agents intentionally added to food or feed as notified to EFSA 22: Suitability of taxonomic units notified to EFSA until March 2025. EFSA J..

[86] European Parliament and Council (2006). Regulation (EC) No 1924/2006 of 20 December 2006 on nutrition and health claims made on foods. Off. J. Eur. Union.

[87] European Parliament and Council (2003). Regulation (EC) No 1829/2003 of 22 September 2003 on genetically modified food and feed. Off. J. Eur. Union.

[88] Consumer Affairs Agency, Government of Japan (2026). For Informed Food Choices. Consumer Affairs Agency, Japan. https://www.caa.go.jp/en/about_us/booklet/assets/pamphlet_en_2026_0010.pdf.

[89] Consumer Affairs Agency, Government of Japan (2020). Foods with Function Claims Notification F680: Nattokinase Sarasara Tsubu. Consumer Affairs Agency, Japan. https://www.fld.caa.go.jp/caaks/s/cssc01/fwccd01?cid=a09F900004sk8DRIAY.

[90] U.S. Food and Drug Administration (2020). GRAS Notice No. 887: Menaquinone-7. U.S. Food and Drug Administration. https://www.hfpappexternal.fda.gov/scripts/fdcc/index.cfm?set=GRASNotices&id=887.

[91] U.S. Food and Drug Administration GRAS Notices. https://www.hfpappexternal.fda.gov/scripts/fdcc/index.cfm?set=GRASNotices.

[92] U.S. Food and Drug Administration FDA 101: Dietary Supplements. https://www.fda.gov/consumers/consumer-updates/fda-101-dietary-supplements.

[93] National Health and Family Planning Commission of the People’s Republic of China (2016). Interpretation of the Announcement on Calcium Alginate and Other New Food Additives. https://www.nhc.gov.cn/zwgk/jdjd/201606/a807d534ce0149f5bc693e12aa602c4c.shtml.

[94] Hubei Provincial Health Commission (2026). DBS42/020—2026 Food Safety Local Standard: Natto Powder. Hubei Provincial Health Commission. https://wjw.hubei.gov.cn/zfxxgk/zc/gkwj/ywh/202602/P020260203609890835991.pdf.

